# Facilitate behavior change among surgeons for infection prevention and stewardship: lessons learned from Asia

**DOI:** 10.1017/ash.2025.10252

**Published:** 2025-12-18

**Authors:** Anucha Apisarnthanarak, Pataravit Rukskul, Linda M. Mundy

**Affiliations:** 1 Division of Infectious Diseases, Thammasat University Hospital, Pratum Thani, Thailand; 2 Division of Neurosurgery, Thammasat University Hospital, Pratum Thani, Thailand; 3 Department of Medicine, Icahn School of Medicine at Mount Sinai/Elmhurst, NY, USA

## Abstract

This commentary informs on key pragmatic contributors to strategic and sustainable surgical IPC and AMS initiatives. Three key recommendations to strengthen and sustain surgical IPC and initiatives are (1) institutional leadership support, (2) a programmatic multidisciplinary implementation plan, and (3) effective communication strategies using motivational interview.

## Commentary

Surgical care environments are complex, high-pressure settings where work flow, technical precision, and time efficiencies contribute to patient outcomes.^1^ Successful surgical infection prevention and control (IPC) programs require implementation of evidence-based practices, up-to-date policies, multidisciplinary collaboration, and dedicated leadership.^2^ Factors categorized as administrative and human behavior influence the uptake, diffusion, and integration of perioperative IPC and antimicrobial stewardship (AMS) practices. This commentary informs on key pragmatic contributors to strategic and sustainable surgical IPC and AMS initiatives. Three key recommendations to strengthen and sustain surgical IPC and initiatives are (1) institutional leadership support, (2) a programmatic multidisciplinary implementation plan, and (3) effective communication strategies.

## Executive, surgical, and IPC leadership support

There are many published guidance recommendations to support innovative IPC quality improvement and sustained practice initiatives that are relevant to perioperative care.^3,4^ At the institutional level, IPC teams generally conduct annual risk assessments to prioritize data-driven ranking of risks and hazards to focus on followed by gain of agreement and resources from the executive leadership team for the plan. For the selected surgical priorities such as risk minimization of medical errors, healthcare-associated infection (HAI), and appropriate medication management, joint institutional support from executive, surgical, IPC, and pharmacy leadership is foundational for the trust necessary to foster commitment, accountability, and results.^3–5^ At the top of the hierarchy of risk controls adopted from industry and occupational health, elimination, substitution, and engineering controls are deemed more effective than administrative and human behavioral controls as they control exposures without significant human interaction.^5,6^ In surgical care areas, elimination, substitution, and engineering controls include but are not limited to single-use equipment, central sterile processing of reusable equipment, electrical control checks of equipment, operating and procedural room standards for temperature, negative air pressure, and humidity, and ongoing substitution of newer, safer equipment when feasible. In contrast, administrative and human factor controls entail significant commitment to policy and practice compliance, work flow, effective communication, mutual purpose agreements, education on new initiatives and standards, training, and psychological safety.^5,6^ System designs and outcome-based feedback can facilitate surgical IPC and AMS initiatives that entail administrative and human factor controls, yet hierarchical surgical team structure, particularly in Asian countries, may influence the psychological safety necessary to address real-time breaches in IPC practices and the adoption of evidence-based AMS practices. Administrative surgical leadership is quintessential for a safe surgical care environment, cultivation of a culture of excellence, inspiring surgical staff, support of quality improvement, and framing solutions for identified issues.^7^

## Surgical IPC and AMS programmatic plans

For new surgical IPC initiative, the following pragmatic six-step approach has been effective at Thammasat University Hospital (TUH), Thailand, after assurance of joint administrative leadership endorsement:

1. Establishment of a multidisciplinary team, with written and agreed to clarification of roles and responsibilities for team members. The team should address the decision-making processes for governance, surveillance, and goals.
2. Identification of the specific surgical IPC or AMS intervention. Evidence-based initiatives from international, national, or regional sources should be acknowledged, yet the proposed intervention should be endorsed by the multidisciplinary team prior to implementation and consideration given to the potential positive and negative impact of the proposal on existing surgical, IPC, and AMS practices.
3. Selection of the appropriate surgical service(s) or unit(s) for the initiative.
4. Track the change over time, facilitators and barriers to the intervention, provide feedback to key stakeholders, and escalate issues as necessary. If the implementation of the defined initiative is successful and sustained, leadership support and resources to address facilitators and barriers should be identified prior to expansion to additional surgical services or units.
5. Disseminate the initiative to other surgical services or units and continue to assess sustainment of the initiative on the original service unit. Consider identification of a surgical champion to lead and inform peer surgeons and providers of transitioning the IPC or AMS initiative into standard practice.
6. Sustainment of the initiative into standard surgical practice is an essential step that requires administrative leadership endorsement to integrates the IPC or AMS initiative into standardized surgical practice.

## Effective communication strategies to support uptake of new surgical IPC and AMS initiatives

In hierarchical cultures such as surgical settings in Asia, administrative and human factor change in perioperative care practices may require a variety of effective communication strategies. A case example of leadership support for a multidisciplinary team’s neurosurgical (NS) IPC and AMS initiative is provided that benefited from targeted use of motivational interview techniques to support dissemination and implementation of the initiative.^10^

**Figure 1.**
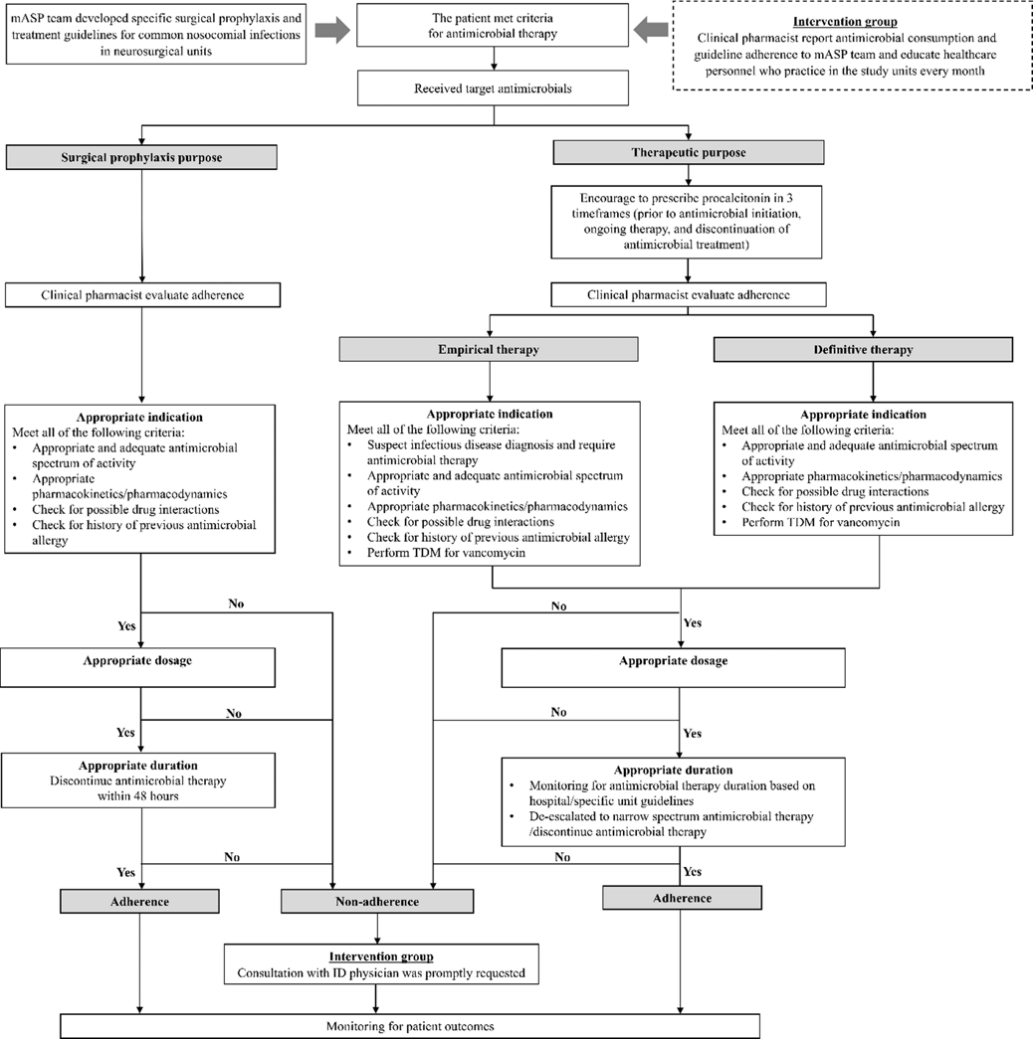
Antimicrobial therapy workflow in the neurosurgical unit at Thammasat University Hospital, Thailand. *Note:* ID, infectious diseases; mASP, multidisciplinary antimicrobial stewardship program; TDM, therapeutic drug monitoring.

After a confirmed outbreak of carbapenem-resistant *Acinetobacter baumannii,* the NS unit providers routinely prescribed a carbapenem agent for preoperative NS cases and initial empiric treatment of suspected HAI. Surveillance data also confirmed a significant increase in extra ventricular drainage (EVD) associated central nervous system HAI attributed to multidrug-resistant (MDR) pathogens. Consensus in joint administrative leadership support of a NS intervention was followed by the formation of a multidisciplinary team comprised of NS physician and nursing staff, an infectious diseases (ID) specialist, an ID AMS pharmacist, and an IPC representative. Seven agreed to NS interventions were 1) use of cefazolin as surgical prophylaxis via a standing order and 24-hour stop, 2) creation of specific antimicrobial guidance for preoperative NS prophylaxis and for treatment of suspected NS HAI,^8^ 3) ID consultancy for all patients prescribed a carbapenem (Figure 1), 4) IPC monitoring of hand hygiene, use of personal protective equipment, and isolation precautions for all MDR-cases, 5) daily chlorhexidine bath for all patients in the NS intensive care unit, 6) incorporation of the EVD-bundle into routine care of patients with EVD devices,^9^ and 7) creation of an AMS algorithm (Figure 1). While the NS leadership fully supported the multipronged interventions, the NS teams complained about the initiative for a standing order, the need for an ID consultant to monitor NS care, the routine use of daily chlorhexidine baths, and the components of an EVD-bundle requiring compliance monitoring.

The joint administrative leadership team was informed of the resistance to the NS IPC and AMS initiative and agreed with the build of communication strategies focused on mutual purpose and optimal health outcomes. One of the multidisciplinary team strategies to enhance uptake and diffusion of the initiate was motivational interview techniques which included use of open-ended questions to evoke thoughts and feelings about the interventions and powerful listening without judgment to the interviewee responses.^10^ One example of a motivational interview for the NS initiative follows:

- *NS leader and ID specialist.* Thank for meeting with us. We guess you have been having some trouble with this surgical IPC initiative.
- *NS staff member:* We are disgusted with it. We have been ordering antibiotics for NS prophylaxis and for treatment for 10–20 years and never had a problem. All of a sudden, the IPC team come up with a new regulation. As far as I concerned, if it isn’t break, don’t fix it. It is a pity the poor residents and fellows who are supposed to unlearn what they have just been taught.
- *NS leader and ID specialist:* You are really frustrated about this. What else bothering you about the new initiatives?
- *NS staff member:* Don’t get me started. All these people telling me what to do. I have got ID fellows telling me when I should and should not prescribe antibiotics. Give me a break!
- *NS leader and ID specialist:* What do you understand about the science behind the initiative?
- *NS staff member:* That is another thing. They keep coming up with a new quality project, it is like a blizzard of them, and a couple years later they change their minds. This is probably just another of those things.
- *NS leader and ID specialist:* We hear you. You are frustrated getting hit by these changes. We really appreciate how open you are being with us. One thing though, can you tell us whether your opposition to our initiative is making thing awkward for your colleagues?
- *NS staff member:* Well, may be a little, especially for our fellows and residents. I guess the hospital epidemiologist, clinical pharmacist, and infection preventionist who are running the initiative are also getting annoyed with us.
- *NS leader and ID specialist:* You seem to be saying that you are a little bit uncomfortable about that.
- *NS staff member:* Well, they are pretty committed to making the changes and they are not totally stupid.
- *NS leader and ID specialist:* Speaking of that, I got some data about the impact of antibiotic stewardship in the NS unit and use of a chlorhexidine bath to reduce MDR-pathogens. I thought you might be interested in this data.
- *NS staff member:* I suppose so.
- *NS leader and ID specialist:* The data suggested that integrating antimicrobial stewardship in the NS unit resulted in a significant reduction in the emergence and transmission of MDR-pathogens. In addition, the use of the daily chlorhexidine bath in ICUs has been used around the world resulting in several reports of reduction in MDR-pathogens in ICU settings. The patients’ populations studied are fairly similar to our NS ICU. Do you know that our incidence of carbapenem-resistant Gram-negative pathogens is still rising, particularly carbapenem-resistant *A. baumannii*?
- *NS staff member:* No, we did not realize that it was still rising. I think it is something to worry about.
- *NS leader and ID specialist*: The hospital accountants are all about making patients happy customers. Transmission of MDR-pathogens can lead to a significant increase in patients’ morbidity and mortality, particularly the older surgical patients with multiple comorbidities that you care for.
- *NS staff:* We hear you. Well, if that is the case, we are going to have to give in sooner.

After this and other motivational interviews, there was less resistance to the NS initiative by the NS team. The interventions led to a successful reduction in carbapenem-resistant *A. baumannii* and a sustained reduction in EVD-associated infections.^8^ While there are various types of communication algorithms to optimize effective team initiatives, this case example of motivational interviewing in an Asian surgical healthcare setting was embedded within the six-step approach to a NS IPC and AMS initiative, and informs on a feasible approach to dissemination and implementation science in surgical settings.

## Lessons learned

### For effective administrative leadership and implementation plans

- Frame proposed changes around patient safety and clinical performance to improve receptivity of the plan. Surgeons prioritize interventions that demonstrably reduce complications, improve workflow efficiency, lower the risk of re-operation, and minimize legal exposure. Presenting interventions as mechanisms to enhance patient outcomes, rather than as administrative mandates, aligns with professional values and fosters intrinsic motivation.
- Early engagement of surgeons in protocol design and implementation further enhances compliance. Involvement in reviewing evidence, offering feedback, and participating in process design fosters a sense of ownership, reducing resistance, and increasing adherence. Similarly, direct and respectful communication—concise, nonemotional, and acknowledging professional expertise—supports collaboration and minimizes confrontation.

### For effective communication

- Use data-driven communication. Surgeons respond most effectively to objective metrics such as infection rates, operating room (OR) delays, and compliance audits, especially when presented alongside peer benchmarking and clear cause–effect relationships. Framing information in terms of outcomes—for example, noting that “surgical-site infection (SSI) rates for procedure X are twice as high when step Y is skipped”—rather than issuing prescriptive criticism depersonalizes the message and reduces defensiveness.
- Peer influence represents another critical lever for change. Surgeons are highly influenced by respected colleagues, particularly senior surgeons or specialty champions. Initiatives such as morbidity and mortality discussions or presentations by surgeon role models create an environment in which new practices are endorsed from within the professional community, enhancing acceptance and sustainability.
- Positive reinforcement is more effective than criticism in sustaining behavioral change. Acknowledging improvements or consistent adherence to protocols—such as noting enhanced OR efficiency or lower SSI rates—encourages continued compliance and strengthens team morale.
- Addressing systemic barriers rather than personality traits is crucial. Surgeons’ behaviors are often influenced by time pressures, workflow inefficiencies, miscommunication, or equipment limitations. Optimizing the surrounding system is generally more effective than attempting to modify individual traits.
